# Radiographic parameters and mechanical complications of articulating vs. non-articulating hip spacers

**DOI:** 10.1007/s00402-026-06347-8

**Published:** 2026-05-20

**Authors:** Julia Lenz, Florian Baumann, Susanne Baertl, Josina Straub, Markus Rupp, Volker Alt, Viola Freigang

**Affiliations:** 1 Department of Trauma Surgery, University Hospital Regensburg, Regensburg, Germany; 2 Department of Trauma Surgery, Universitätsklinikum Gießen und Marburg, Giessen, Germany; 3 Hochschule Landshut, Landshut, Germany

**Keywords:** Periprosthetic joint infection, Hip spacer, Articulating spacer, Non-articulating spacer, Two-stage revision, Mechanical complications

## Abstract

**Background:**

Periprosthetic joint infection (PJI) is a serious complication of total hip arthroplasty. Standard treatment for PJI is a two-stage revision surgery with antibiotic-loaded cement spacers to control infection and provide temporary joint stability. This study compares the radiological outcomes and complication rates between articulating and non-articulating hip spacers in patients undergoing treatment for PJI and native joint infections.

**Methods:**

We retrospectively reviewed 71 hip spacers (34 articulating, 37 non-articulating) in 38 patients treated between 2004 and 2022. Data on leg length discrepancy, femoral offset, infection control, and mechanical complications were obtained. For infection analysis, only patients treated exclusively with one spacer type were included. After excluding eight patients with mixed spacer types (29 spacers), 30 patients with 42 spacers were included in this subgroup.

**Results:**

Articulating spacers were significantly better at preserving leg length, with a mean discrepancy of -3.7 mm compared to -16.9 mm for non-articulating spacers (*p* = 0.025). However, non-articulating spacers maintained femoral offset (1.1 vs. 0.7, *p* < 0.001) closer to physiological offset. The rate of mechanical complications was higher in the articulating spacer group, with spacer dislocations occurring in 45% of cases compared to 10% in the non-articulating group (*p* = 0.015). There was no difference regarding infection control between both groups.

**Conclusion:**

In this exploratory cohort, non-articulating spacers were associated with fewer mechanical complications, whereas articulating spacers demonstrated better leg length preservation. Given the exploratory nature of this study and its methodological limitations, the observed differences between spacer types should be interpreted cautiously and considered associative rather than causal.

## Introduction

Periprosthetic joint infection (PJI) is a serious complication after total hip arthroplasty. Management of PJI requires a highly coordinated and thoroughly planned treatment strategy due to the significant morbidity and potential for relapse [1].

Classification systems for PJI may guide surgical treatment decisions. According to the European Bone and Joint Infection Society (EBJIS), PJI can be classified in acute (postoperative and hematogenous) and chronic infections based on symptom onset [2, 3]. The PJI-TNM classification system categorizes infection by its tissue and implant conditions (T), non-human cells (N), and the morbidity of the patient (M), offering a more detailed assessment to tailor treatment [4].

Among available treatment options, two-stage revision has emerged as the gold standard for managing chronic PJI of the hip [5, 6]. This approach involves complete removal of infected prosthetic components and associated bone cement. Extensive debridement of the affected tissue is essential to eradicate infection [7–9]. After debridement, an antibiotic-impregnated cement spacer may deliver high local concentrations of antibiotics directly to the infection site. Prior studies have shown significantly higher infection clearance rates for highly concentrated local antibiotics [10]. Studies have reported infection eradication rates of up to 90% when using these spacers in two-stage revisions [5, 7, 11].

A key benefit of antibiotic-loaded spacers is their ability to achieve high local antibiotic concentrations without significantly increasing systemic levels. Thereby the risk of antibiotic-related systemic side effects, such as nephrotoxicity or gastrointestinal disturbances may be reduced [12, 13]. In addition to their antibiotic delivery function, these spacers play an essential role in maintaining the joint space, minimizing dead space after implant removal, and providing temporary mechanical stability to the affected joint. This may prevent soft tissue contracture and facilitates later re-implantation of the definitive prosthesis [14].

**Fig. 1:**
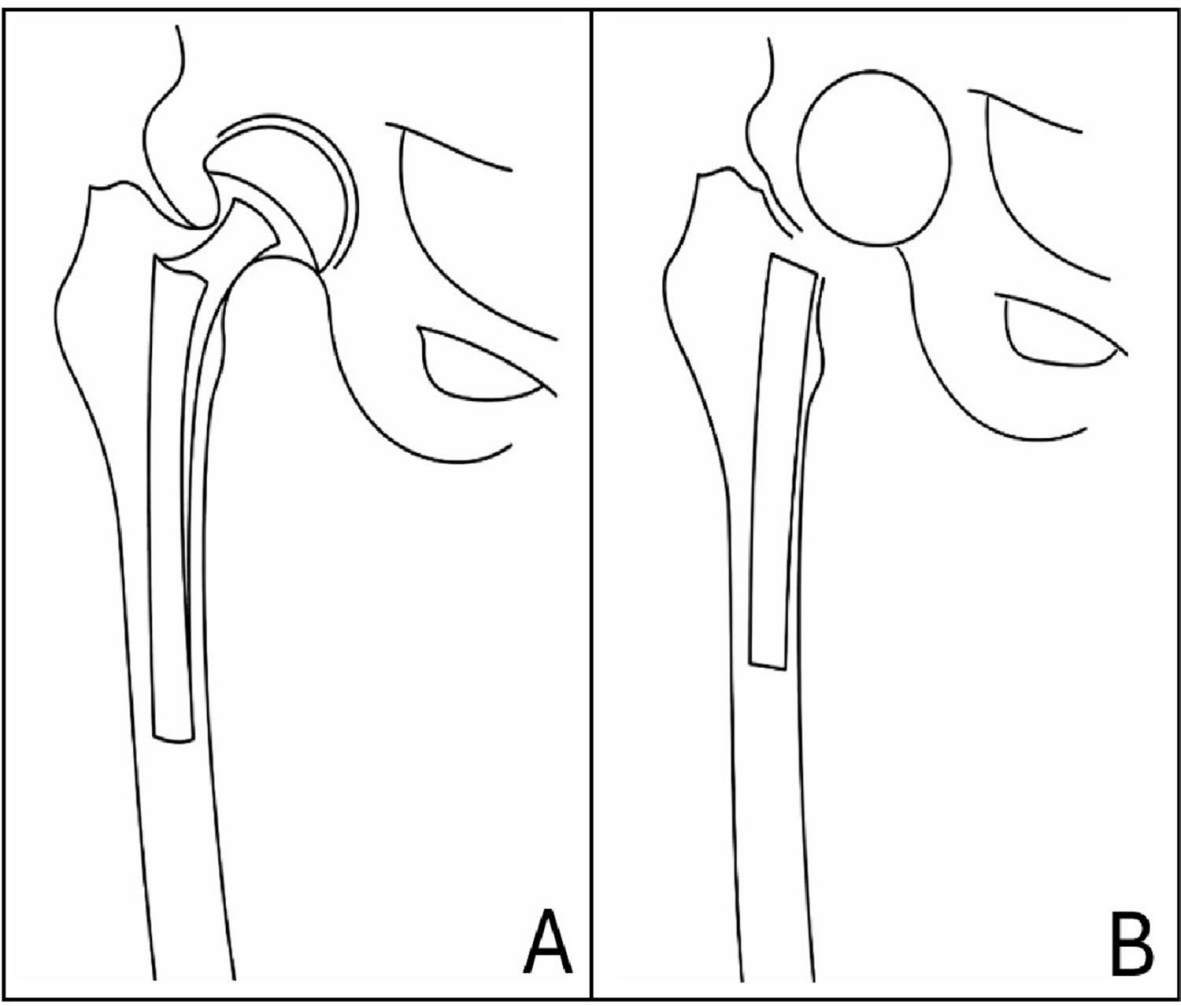
Articulating (**A**) and non-articulating (**B**) spacer design

Hip spacers can be classified into two main types: articulating and non-articulating spacers (Fig. 1). Non-articulating spacers are typically composed of a large ball of antibiotic-impregnated cement placed within the acetabulum, while a metal rod covered with antibiotic cement is inserted into the femoral canal. There is no direct articulation between the femoral and acetabular components, which reduces weight carrying capacity of the leg but may still provide adequate infection control [15]. Articulating spacers, on the other hand, more closely mimic the structure of a hemiarthroplasty. They consist of a monobloc cement design that includes both a femoral and acetabular component, which can articulate and transmit load bearing forces. These spacers are often reinforced with a metal rod within the femoral part to provide additional mechanical stability. By preserving the distance between the femoral head and acetabulum, articulating spacers load transmission to the acetabulum, potentially contributing to improved patient mobility and functional outcomes during the interim period before definitive re-implantation [16].

Despite the theoretical advantages of articulating spacers in terms of mobility and stability, there is a paucity of clinical and radiological data comparing efficacy of non-articulating spacers, particularly in terms of outcomes such as infection recurrence, functional recovery, and complications.

The aim of this study was to assess the radiological characteristics and mid-term clinical outcomes of articulating versus non-articulating hip spacers in patients with hip joint or peri-prosthetic hip infections.

We hypothesized that articulating spacers would demonstrate superior leg length preservation, whereas non-articulating spacers would show improved offset maintenance and fewer mechanical complications.

## Methods

### Study population and patient characteristics

**Fig. 2:**
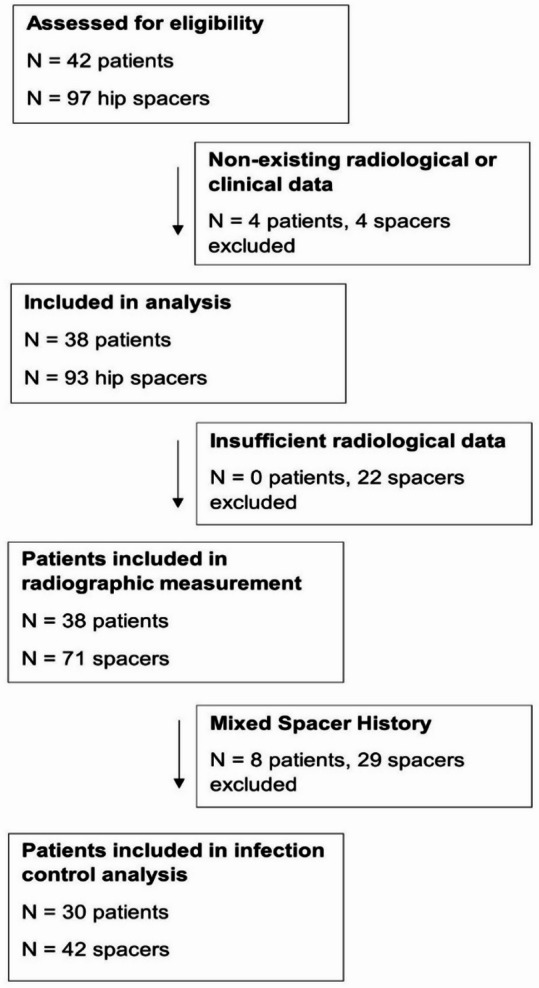
CONSORT flow chart

This single-center retrospective study evaluates the radiographic outcomes of patients with antibiotic-loaded cement spacers for PJI after total hip arthroplasty and native hip infection. The patients underwent two- or multiple-stage revisions between 2004 and 2022. Inclusion criteria for the study consisted of hip joint or prosthesis infections, followed by spacer implantation, with at least one radiological follow-up of the pelvis. The definition of periprosthetic joint infection (PJI) and native joint infections followed the consensus criteria set forth by the European Bone and Joint Infection Society (EBJIS) [2, 17]. Based on these criteria, we enrolled a total of 97 hip spacers in 42 patients (see Fig. 2). Four patients were excluded due to missing radiological or clinical data. 22 spacers were excluded from analysis due to insufficient radiological data leaving 71 spacers in 38 patients for radiographic analysis.

For the infection control analysis, the inclusion criteria were refined to encompass only those patients who had received a single type of spacer - either articulating or non-articulating - throughout the entire course of treatment. Patients who underwent mixed spacer application, defined as sequential implantation of both articulating and non-articulating spacers during their treatment course, were excluded from this analysis. Accordingly, eight patients, involving a total of 29 spacers, were removed. As a result, the infection control subgroup comprised 30 patients with 42 spacers.

### Surgical protocol

In cases of native joint infections unresponsive to debridement or in the presence of osteomyelitis, a resection arthroplasty followed by temporary spacer placement was performed as a salvage measure.

In cases of prosthesis infection, within a thorough debridement, we removed all prosthetic components, foreign materials, and bone cement.

In both cases we collected three to five deep tissue samples from the soft tissue, the cup, and the femoral medullary canal for histopathological and microbiological analysis. In addition, the arthroplasty components were sent for sonication. Polymethylmethacrylate (PMMA) cement was prepared and impregnated with antimicrobial agents, following the protocol described by Kuehn et al. [8]. Non-articulating spacers were made from antibiotic-loaded cement, using a cement ball to minimize intra-articular dead space. We fabricated articulating hip spacers using size-adapted spacer moulds (femoral size ranging from 6.25 to 17.5 mm, with head diameters of 44 to 58 mm). The two-part silicone spacer mold (Implantcast Inc., Germany) was filled with cement, and a metal rod was placed centrally to serve as a rigid core. The femoral stem was then topped with a large head of bone cement using a spherical mold.

### Postoperative protocol

Postoperative management included suction drainage for 48 h. Standard antibiotic therapy consisted of intravenous antibiotics for two to six weeks, followed by oral antibiotics for four to twelve weeks. Antibiotics were initially broad-spectrum, adjusted to targeted therapy based on intraoperative culture results. Laboratory values and clinical symptoms, particularly wound drainage lasting more than three weeks, were closely monitored to guide the need for additional revisions. If required, the same debridement and spacer implantation protocol was followed. Re-implantation of the definitive prosthesis was considered after clinical symptoms resolved and laboratory findings remained normal for at least six weeks. During re-implantation, three to five deep tissue samples were again taken for histopathology and cultures. The postoperative protocol was identical, with drainage for 48 h and continuation of antibiotics. All patients received anticoagulation prophylaxis according to guidelines and individual risk profiles. Follow-up assessments, including x-rays, clinical examination, and blood testing, were conducted at six weeks, 12 weeks, six months, one year, and two years. Healing of infection was defined by the absence of clinical, radiological, and laboratory signs of recurrence of infection at the final follow-up.

### Patient demographic and clinical data

Patient demographic and clinical data were retrospectively extracted from our hospital’s clinical information system, including surgical reports and discharge summaries. Collected demographic variables included patient age, sex, and follow-up duration (in months). Clinical variables recorded were the side of the implanted spacer (right or left), type of hip infection (prosthetic or septic arthritis), recurrence of prosthetic joint infection (categorized as yes, no, or infection of a native joint), and the number of revision surgeries performed from the time of spacer implantation until the definitive hip prosthesis was implanted. Additionally, the time between spacer implantation and hip re-implantation was documented.

Infection-related variables were classified according to the TNM system, including the extent of infection (T), the degree of tissue involvement (N), and the microbial load (M) [4]. The infection status at the last follow-up was recorded as healed, not healed, or unclear (if the follow-up was less than three months without infection). Spacer type (articulating or non-articulating) was documented. Patients were categorized into two groups: those who had received only articulating spacers and those who had received only non-articulating spacers. Patients having received both spacer types were excluded for the infection-control analysis.

Radiological and surgical complications consisting of spacer dislocation, spacer fracture, femoral fracture requiring surgery, acetabular fracture requiring surgery, and hematoma/seroma requiring evacuation were assessed based on postoperative reports and follow-up imaging. These data were validated through a careful review of operative records and radiological reports.

### Radiological analysis

**Fig. 3:**
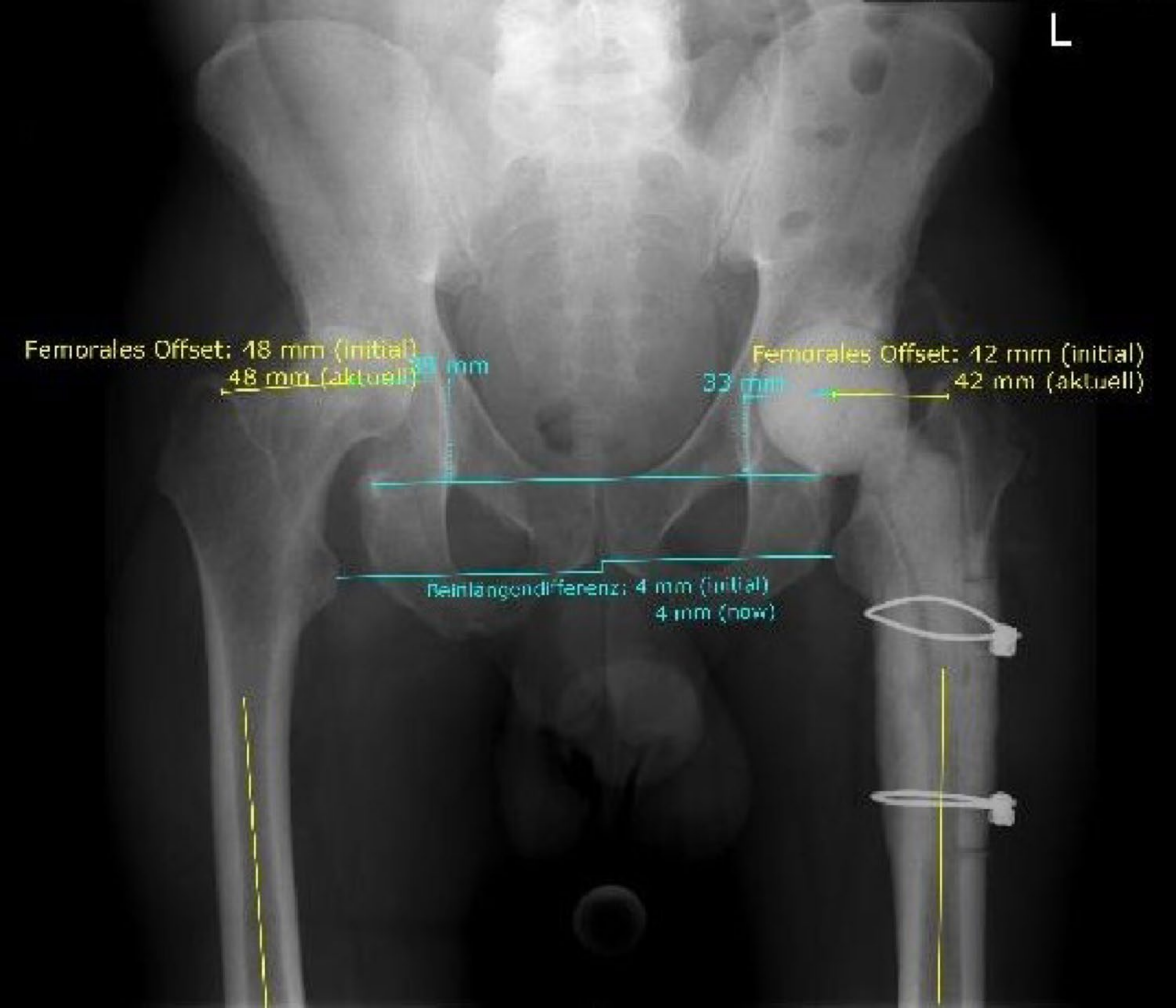
Radiographs with measurement of the femoral offset and leg length

All postoperative radiographs were acquired in standardized anteroposterior pelvic views with the patient in a supine position. Femoral offset and leg length were evaluated according to the method outlined by Flecher et al. [18] (see Fig. 3) using the software mediCAD (Hectec, Inc, Germany) with scaling based on a 25 mm calibration marker placed at the level of the greater trochanter [19, 20].

We measured the leg length discrepancy as the difference in distance between the trans-teardrop line and the center of each femur’s lesser trochanter. Femoral offset was defined as the perpendicular distance from the femoral axis to the center of rotation of the cup.

For each patient, femoral offset was measured and directly compared with the contralateral non-operated hip. The femoral offset ratio was calculated as the quotient of the offset of the spacer-bearing hip divided by the offset of the contralateral, native hip.

Spacer dislocation was defined as a displacement of the spacer head relative to the acetabular cavity on standard AP radiographs, regardless of whether the spacer was articulating or non-articulating.

### Data analyses

Radiographic parameters were analyzed on a per-spacer basis, as each spacer represents an individual mechanical construct with distinct geometry and positioning. In contrast, all clinical outcomes and complications were analyzed on a per-patient basis.

Data analyses were performed using the software package SPSS (Version 28, SPSS Inc., Chicago, Illinois). Distribution of continuous variables was assessed using the Shapiro–Wilk test and inspection of histograms. Given the small sample size and non-normal distribution of several parameters, nonparametric testing (Mann–Whitney U test) was used where appropriate. Effect sizes are presented with 95% confidence intervals.

For the comparison of mean values, the independent *t*-test was used. For ordinal data, the Chi-square test was used. For non-normally distributed data, the Mann-Whitney U test was used. Unless otherwise stated, descriptive data are given as means ± standard deviation. The level of significance was at *p* < 0.05 for all tests.

### Ethical standard

The Ethics Committee at the University of Regensburg granted approval for this study in June 2021 (Institutional Review Board Number 21–2434 − 104). All procedures adhered to the ethical standards outlined in the Declaration of Helsinki (1964). The need for informed consent was waived by the Ethics Committee due to the retrospective nature of the study.

### Demographics

A total of 71 hip spacers in 38 patients were eligible for radiographic analysis. Of these, 34 were articulating and 37 were non-articulating. Among all patients, 20 received only articulating spacers, 10 only non-articulating spacers, and eight had both types at different treatment stages. 14 patients contributed more than one spacer to the analysis.

The mean age of the patients was 66.2 ± 12.3 years. The average time from spacer implantation to reimplantation was 2.7 ± 2.5 months. Follow-up duration was highly variable, with a median of 4.0 months (IQR 1.0–17.0 months, range: 0–101 months). The groups of articulating spacers and non-articulating spacers were demographically comparable (see Table 1). Two patients presented with bilateral infected hip prostheses and were treated with mixed spacers.

**Table 1:** Baseline characteristics of articulating vs. non-articulating spacer groups

Variable	Articulating only (*n* = 20)	Non-articulating only (*n* = 10)	*p*-value
Age, years (mean ± SD)	68.4 ± 12.2	69.9 ± 9.8	0.826
Sex, male/female (n)	9/11	7/3	0.260
Follow-up, months (median, IQR)	3.5 (1.0–11.5)	13.5 (2.75–18.75)	0.442
Revision surgeries after spacer implantation (mean ± SD)	1.6 ± 1.7	0.7 ± 1.3	0.302

**Table 2:** Distribution of infection types and spacer usage in hip infection treatment

Infection type	Number of patients (percentage)	Spacer	
		Articulating	Non-articulating
Periprosthetic joint infection	26 (86.7%)	19	7
Native septic arthritis	4 (13.3%)	1	3

Table 2 shows the distribution of infection types and spacer usage.

**Fig. 4:**
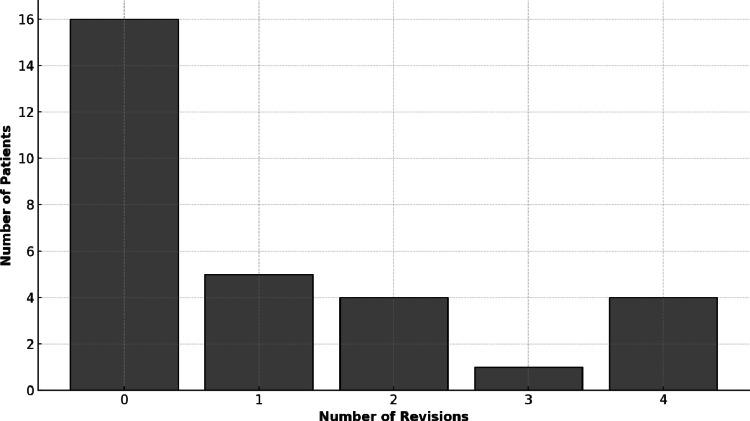
Number of revision surgeries performed between spacer implantation and definitive hip prosthesis implantation

Hip prosthesis reimplantation was performed in 21 patients. The mean time between spacer implantation and hip reimplantation was 2.7 ± 2.4 months (range, 0–8 months). The number of revision surgeries performed between spacer implantation and definitive hip prosthesis implantation are shown in Fig. 4.

A descriptive comparison between the articulating and non-articulating groups showed no statistically significant differences in baseline characteristics. Age was comparable between groups (*p* = 0.826), as was follow-up duration (*p* = 0.106). Likewise, no significant difference was observed regarding revision burden after spacer implantation (*p* = 0.302).

## Results

According to the TNM classification system, most patients with PJI (20 patients) had a stable standard implant without a soft tissue defect (T0a) (see Table 3). Regarding the microbial load, 11 patients had a mature biofilm (previously classified as chronic) with infections caused by “non-difficult-to-treat” bacteria, 6 patients had a polymicrobial biofilm, and 6 patients had a mature biofilm with a culture-negative infection. Pre-existing comorbidities varied widely across patients.

**Table 3:** Distribution of patients with PJI according to TNM classification and spacer group

			Total *N* = 26	Spacer - articulation	
				Yes *N* = 19	No *N* = 7
*“Tissue and implant conditions” - soft tissues and implant*					
T0	a	Stable standard implant without soft tissue defect	20	14	6
T1	a	Loosened standard implant without soft tissue defect	4	4	0
T2	a	Severe soft tissue damage with standard implant	2	1	1
*“Non-human cells” - bacteria and fungi*					
N0	a	Immature biofilm (previously: acute), directly postoperative	1	1	0
N1	a	Mature biofilm (previously: chronic), infection with “non-difficult-to-treat” bacteria	11	7	4
	b	Mature biofilm (previously: chronic), culture-negative infection	6	4	2
N2	a	Mature biofilm (previously: chronic), infection with “difficult-to-treat” bacteria	2	2	0
	b	Mature biofilm (previously: chronic), polymicrobial infection	6	5	1
*“Morbidity” – pre-existing conditions*					
M0		Not or only slightly compromised (Charlson Comorbidity Index: 0–1)	7	6	1
M1		Moderately compromised (Charlson Comorbidity Index: 2–3)	8	5	3
M2		Severely compromised (Charlson Comorbidity Index: 4–5)	8	5	3
M3	c	Patient is not eligible for an operation	3	3	0

Non-reported values are omitted for clarity and equal to 0. Only patients with periprosthetic joint infection (PJI) are included [4]

At the final follow-up, 16 patients were classified as “healed infections”, 4 patients were not healed, and 10 patients had less than three months of follow-up but showed no signs of infection at the last available follow-up. No detectable difference in infection control was observed between spacer groups in this small cohort.

Complications were noted in several patients, with varying rates between spacer types (see Table 4).

We found a significant difference regarding the rate of spacer dislocations. Spacer dislocations were more common in the articulating spacer group, affecting 45% of patients (9 of 20 patients), compared to 10% of patients (1 of 10 patients) in the non-articulating group.

Spacer dislocation was defined as any displacement of the spacer relative to the acetabular cavity on standard anteroposterior radiographs, applicable to both articulating and non-articulating spacers. Due to the retrospective study design, detailed information regarding the exact timing, recurrence pattern, and management of dislocation events could not be consistently reconstructed.

There was no significant difference regarding the number of spacer fractures, femoral fractures, acetabular fractures, or hematoma.

**Table 4:** Complications with the need for revision surgery

Complication	Total	Spacer		*p*-value
		Articulating *N* = 20	Non-articulating *N* = 10	
Spacer dislocation	10	9 (45%)	1 (10%)	0.015*
Spacer fracture	2	2 (10%)	0 (0%)	0.081
Femoral fracture	11	9 (45%)	2 (20%)	0.084
Acetabular fracture	2	2 (10%)	0 (0%)	0.081
Hematoma/seroma	8	6 (30%)	2 (20%)	0.281

The values are given as the number of cases in the cohorts with only one spacer design, with the percentage in parentheses. * significant, <0.05

### Radiological analysis

**Fig. 5:**
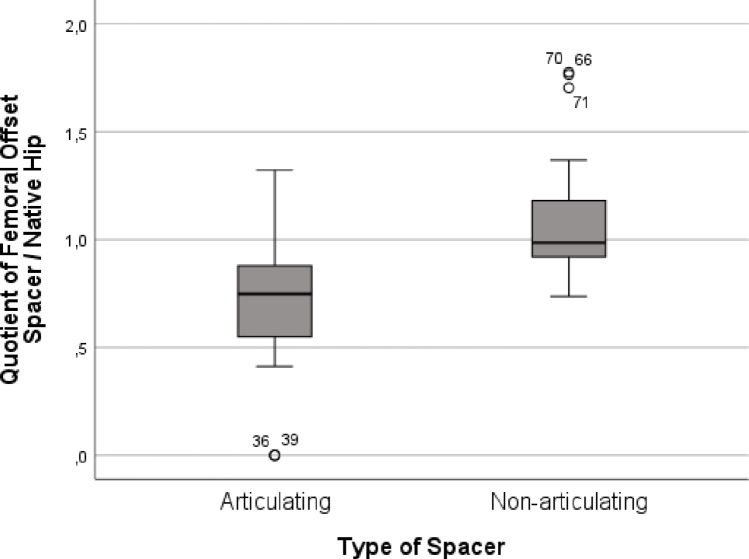
Leg length difference in mm between spacer and native hip. Negative values indicate leg shortening. Median values varied with statistical significance (*p* = 0.020)

The median leg length discrepancy was significantly smaller in the articulating group (-0.4 mm IQR − 13.0–3.5) than in the non-articulating group (-31.5 mm IQR − 43.2–2.2). The Hodges–Lehmann estimate of the median difference was 21.6 mm (95% CI 3.2 to 33.7 mm; *p* = 0.020) (Fig. 5).

**Fig. 6:**
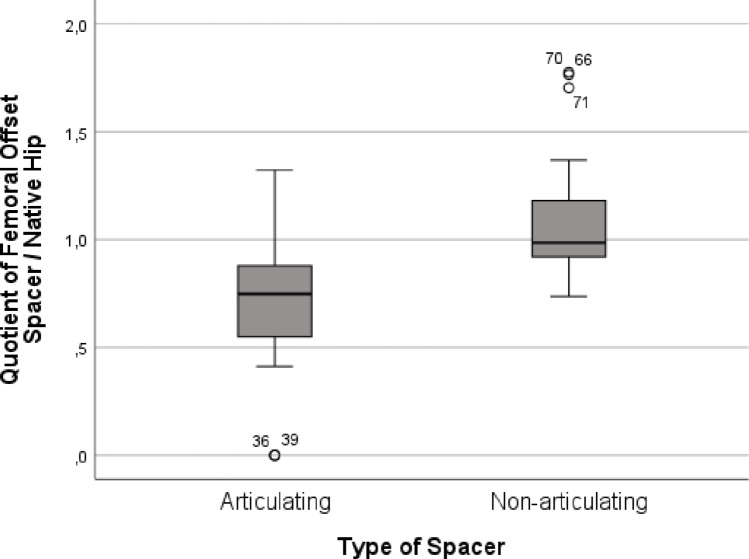
Quotient of femoral offset of spacer/native hip. Values varied with statistical significance (*p* < 0.001)

The ratio of femoral offset between the spacer and the native hip was 0.7 ± 0.3 for articulating spacers and 1.1 ± 0.3 for non-articulating spacers (see Fig. 6), with the difference also being statistically significant (*p* < 0.001).

## Discussion

This study provides new insights regarding radiographic outcome of articulating and non-articulating spacers in treatment of PJI. Given the retrospective design and limited sample size, the present study should be regarded as exploratory and hypothesis-generating rather than confirmatory. We found no difference in infection eradication rate between the two spacer types. Articulating spacers can maintain leg length, whereas non-articulating spacers may provide preservation of femoral offset.

### Leg length and offset preservation

A key finding of this study is that articulating spacers were significantly better at preserving leg length compared to non-articulating spacers. The smaller leg length discrepancy observed with articulating spacers aligns with their design, which allows for more anatomical restoration of the joint, offering improved mobility during the interim period before re-implantation [21]. Patients who prioritize mobility and comfort during the spacer phase may also benefit from a reduced risk of gait disturbances and functional limitations. Pinsornsak et al. reported no significant differences in functional outcomes between handmade and pre-fabricated spacers, but they emphasized the importance of maintaining leg length and offset to minimize mechanical complications [22].

Despite better leg length preservation, articulating spacers were inferior in maintaining femoral offset compared to non-articulating spacers. Offset preservation is critical for ensuring abductor function and joint stability. Reduced femoral offset, as seen with articulating spacers, can compromise muscle tension and contribute to instability, potentially leading to mechanical complications after re-implantation. Non-articulating spacers provided increased offset maintenance, which may help prevent complications related to joint instability. Jones et al. highlighted the need to focus on offset restoration and femoral fixation to prevent mechanical complications such as dislocation [23].

It is important to note that the higher offset in non-articulating spacers we observed should be interpreted with caution. If the femur is positioned laterally without adequate tension or appropriate length, the offset may appear increased. This may compromise accurate measurement of the offset. Offset alone, without proper alignment and muscle tension, fails to provide the necessary biomechanical benefits. This nuance underscores the need for a balanced approach, prioritizing both offset preservation and the restoration of functional parameters, rather than relying solely on offset as a surrogate for stability.

Although non-articulating spacers demonstrated a higher femoral offset ratio compared to articulating spacers, an offset ratio exceeding 1.0 indicates lateralization relative to the native hip rather than anatomical restoration. Over-lateralization may alter abductor tension, joint reaction forces, and acetabular loading patterns. Therefore, greater offset preservation in this cohort should not be interpreted as inherently biomechanically superior but rather as a radiographic observation whose clinical implications remain uncertain.

### Mechanical complications

Articulating spacers demonstrated an increased overall rate of mechanical complications, with statistical significance observed in the incidence of spacer dislocations. Spacer dislocations were observed in 45% of the articulating group. This finding is concordant with the literature. The dislocation rate in literature ranges from 0% to 41% and is the most frequently reported complication in spacer therapy [24–27]. In our study, the dislocation rate in the articulating group was on the higher end of this spectrum and may reflect multiple contributing factors reported in the literature, including reduced offset, soft tissue compromise, acetabular defects, and spacer positioning, which can lead to abductor dysfunction and instability. Several studies, including those by Leunig et al. and Erivan et al., reported that smaller offsets are associated with a higher risk of dislocation [24, 28]. Bori et al. suggest that acetabular bone defects, spacer positioning, and muscular insufficiency all contribute to spacer instability and subsequent dislocation [29]. These factors may have played a role in the high dislocation rate observed in our articulating group. These findings represent associations rather than causal effects, and potential contributing factors such as reduced femoral offset, compromised soft tissues, or bone loss can only be discussed as possible explanations. No within-cohort analysis was performed to directly assess the association between femoral offset and dislocation risk.

Spacer fractures were another mechanical complication noted in our study. Spacer fractures were reported in 10% of patients with articulating spacers, compared to 0% in the non-articulating group. This finding echoes results from other studies. Sambri et al. found spacer fractures in 3.5% of cases, mainly in pre-fabricated cement spacers [27, 30]. The use of a metallic endoskeleton as used in our study may contribute to the relatively low fracture rates. Other studies have shown improved spacer stability and reduced fracture risk with the use of metallic reinforcement [23]. Even with this reinforcement, articulating spacers were more prone to result in spacer fractures. This may be due to the increased mechanical stress with partial load bearing [31].

### Hematoma and seroma formation

Hematoma or seroma formation requiring evacuation was also more frequent in the articulating and mixed groups compared to the non-articulating group. The formation of these fluid collections may be associated with increased soft tissue manipulation during spacer implantation. This complication has been noted in other studies, such as by Anagnostakos et al., who observed a correlation between mechanical complications and increased spacer handling during surgery [32]. Proper surgical technique and careful handling of soft tissue is essential to minimize the risk of hematoma or seroma formation, which can further complicate recovery.

### Patient selection and functional considerations

The choice of spacer type should be based on the patient’s functional needs, comorbidities, and ability to tolerate potential complications. Articulating spacers may be preferable for more active patients who prioritize mobility during the interim period before re-implantation. However, the higher risk of mechanical complications, particularly dislocation, must be weighed against the potential benefits of improved leg length restoration. Non-articulating spacers, while less effective in maintaining leg length, provide fewer mechanical complications, making them more suitable for patients with lower activity levels or those at higher risk of dislocation. As noted by Erivan et al., patients with compromised health or significant soft tissue loss may benefit from a more stable, non-articulating spacer design [28].

### Limitations

This study has several limitations, including its retrospective design, relatively small sample size, and the inclusion period, which may reflect evolving surgical practices. However, all procedures were performed by surgeons with comparable levels of experience, minimizing the risk of performance bias. Spacer selection was based on individual surgeon judgment without predefined criteria. Due to the retrospective study design, the specific rationale underlying spacer choice could not be reliably reconstructed. Furthermore, the spacer design within the cohorts was not standardized, particularly among non-articulating spacers. A structured assessment of indication criteria would require a prospective study design.

Other factors, such as resistance profiles of the pathogens, were not considered in this analysis, despite their potential influence on re-infection rates. Moreover, we cannot draw conclusions about whether the choice of spacer may have an impact on the implant configuration in re-implantation. Further studies may investigate if articulating spacers may lead to spacer-induced bone defects.

An additional limitation relates to follow-up duration in the infection-control analysis. Ten patients had less than three months of follow-up at the time of final assessment and were classified as not infected based on the absence of clinical, laboratory, or radiographic signs at last contact. However, periprosthetic joint infection recurrence may occur beyond this time frame. Therefore, infection eradication rates reported in this cohort should be interpreted cautiously, as limited follow-up may underestimate late failures.

While all measurements were performed using scaled radiographs, we acknowledge that minor variations in patient positioning could influence radiographic parameters. Furthermore, patients with native hip infections were included, which could have altered outcomes.

Radiographic parameters were analyzed per spacer, as each spacer represents an individual mechanical construct with distinct geometry and positioning. However, we acknowledge that in patients who received more than one spacer, measurements may not be fully statistically independent due to shared anatomical and biological factors. Given the limited sample size, clustered or mixed-effects modeling was not performed. Therefore, radiographic comparisons should be interpreted as exploratory and descriptive.

Another limitation relates to the inclusion of both PJI and native septic arthritis in the radiographic analysis. Although spacer implantation follows similar biomechanical principles in both scenarios, the underlying anatomical and biological environments differ substantially. Patients undergoing explantation of a prosthesis may present with bone defects, altered acetabular geometry, or compromised abductor function, whereas native joint infections may involve different structural conditions. These differences may influence radiographic parameters independently of spacer design. Consequently, radiographic comparisons between spacer types may be partially confounded by heterogeneity of underlying pathology.

Although patients with native septic arthritis and periprosthetic joint infection were analyzed together for radiographic evaluation, clinical outcomes were assessed at the patient level. Given the small number of native joint infections, separate subgroup or multivariable analyses would have been underpowered and of limited interpretability.

Our study did not investigate patient-reported outcome while the spacer was in situ. The spacer design may have implications on the patient’s mobility and health-related quality of life.

Given the retrospective design and absence of predefined criteria for spacer selection, the differences observed between articulating and non-articulating spacers cannot be interpreted as causal or design-related effects. Rather, these findings represent associations within a heterogeneous clinical cohort and may be influenced by confounding factors such as patient condition, bone loss, soft tissue status, or surgeon preference. Therefore, the results should be regarded as exploratory and hypothesis-generating.

## Conclusion

In this exploratory cohort, non-articulating spacers were associated with fewer mechanical complications, whereas articulating spacers demonstrated better leg length preservation. Spacer selection should be tailored to the individual patient, weighing the benefits of interim mobility against the risk of mechanical failure in the context of two-stage revision for PJI and native hip joint infections.

## Data Availability

Data is available from the corresponding author upon reasonable request.
